# Tissue-Specific Genomic Evolution Despite Shared MED12 Mutations in Benign Tumors

**DOI:** 10.3390/jcm14207325

**Published:** 2025-10-16

**Authors:** Jeong Namkung, Sang Ho Park, Ayoung Hwang, Hae Seo, Jooyoung Park, Moonyoung Lee, Hyunkyung Kim, Jungmin Choi, Jae Yen Song

**Affiliations:** 1 Department of Obstetrics and Gynecology, Eunpyeong St. Mary’s Hospital, College of Medicine, The Catholic University of Korea, 1021 Tongil-ro, Eunpyeong-gu, Seoul 03312, Republic of Korea; rossa@catholic.ac.kr; 2 Clinical Research Laboratory, Uijeongbu St. Mary’s Hoapital, The Catholic University of Korea, 271 Cheonbo-ro, Uijeongbu-si 11765, Gyeonggi-do, Republic of Korea; phika77@naver.com; 3 Department of Biomedical Sciences, Korea University College of Medicine, 73 Goryeodae-ro, Seongbuk-gu, Seoul 02841, Republic of Korea; ayounghwang@korea.ac.kr (A.H.); haeseo@korea.ac.kr (H.S.); pjy0722@korea.ac.kr (J.P.); moonyoung_lee@korea.ac.kr (M.L.); jungminchoi@korea.ac.kr (J.C.); 4 Department of Obstetrics and Gynecology, Uijeongbu St. Mary’s Hospital, College of Medicine, The Catholic University of Korea, 271 Cheonbo-ro, Uijeongbu-si 11765, Gyeonggi-do, Republic of Korea; hyuna17@catholic.ac.kr; 5 Department of Obstetrics and Gynecology, Seoul St. Mary’s Hospital, College of Medicine, The Catholic University of Korea, 222 Banpo-daero, Seocho-gu, Seoul 06591, Republic of Korea

**Keywords:** uterine leiomyoma, breast fibroadenoma, MED12 mutation, whole-exome sequencing

## Abstract

**Background/Objectives**: Uterine leiomyomas (ULs) and breast fibroadenomas (FAs) are the most common benign tumors in women, both arising in hormone-responsive mesenchymal tissues and often co-occurring during reproductive years. Despite their shared hormonal sensitivity and frequent MED12 mutations, their downstream molecular evolution remains poorly characterized. This study aimed to investigate whether ULs and FAs, though initiated by similar genetic alterations, diverge in their oncogenic trajectories, thereby addressing the molecular basis for their distinct clinical behaviors. **Methods**: We performed whole-exome sequencing (WES) on 15 uterine leiomyomas and 7 publicly available fibroadenomas with matched normal controls to compare somatic mutations, copy number alterations (CNAs), and mutational signatures. All UL samples were derived from Korean patients who underwent surgical treatment at a tertiary hospital between 2017 and 2019. Somatic variants were analyzed using MuTect2 and Strelka2. FACETS was used to estimate copy number changes in individual samples, and GISTIC2 to identify recurrent and statistically significant copy number alterations across patient cohorts. Mutational processes were inferred using SigProfiler. Microsatellite instability status was determined with MSIsensor2. The study was approved by the Institutional Review Board (UC17SNSI0092). **Results**: Comparative whole-exome sequencing of 15 ULs and 7 FAs from matched tumor-normal samples in an East Asian cohort confirmed both tumor types harbor identical *MED12* p.G44D mutations, establishing shared molecular initiation. However, post-initiation evolution diverged dramatically: ULs exhibited chromosomal instability with 15 copy number amplifications, 6 of which affected oncogenes, but relatively modest point mutations. In contrast, FAs remained chromosomally stable but hypermutated, harboring 1.47× more variants than ULs despite lower tumor purity. Notably, one histologically benign FA harbored multiple loss-of-function mutations plus an *EGFR* gain-of-function mutation typically associated with malignant breast cancer, challenging traditional benign-malignant classifications. **Conclusions**: Despite sharing a common initiating mutation in MED12, ULs and FAs evolve through fundamentally distinct genomic pathways. UL evolves through chromosomal instability, whereas FA evolves through a mutator phenotype, with important implications for understanding tumor biology and molecular-based risk stratification. These findings support a paradigm of tissue-specific oncogenic evolution and underscore the potential clinical utility of genomic profiling in distinguishing benign tumors with atypical molecular features.

## 1. Introduction

Benign tumors arising in hormone-responsive tissues, uterine leiomyomas (ULs) and breast fibroadenomas (FAs), are remarkably common and affect women at different life stages. They impose a combined public health burden ranging from heavy menstrual bleeding and infertility to diagnostic anxiety and surgery. Epidemiological evidence further supports a clinical association between these two conditions: a population-based study using the South Korean National Health Insurance database found that women with symptomatic ULs had a significantly higher prevalence of benign breast diseases, including FAs, compared to those without ULs [1]. Both lesions share a striking molecular convergence on recurrent MED12 mutations yet diverge sharply in downstream genomic architecture and clinical behavior. Parsing this “same trigger, different tissues” paradigm offers a unique window into how estrogen- and progesterone-driven tumorigenesis is sculpted by local mesenchymal ecosystems. Here, we present the head-to-head genomic comparison of ULs and FAs, aiming to define tissue-specific evolutionary trajectories that follow a common initiating event.

ULs are the most prevalent solid tumors of the female pelvis, detectable in up to 77% of women by age 50 and constituting the leading indication for hysterectomy worldwide [2,3,4]. Symptomatic cases manifest with heavy menstrual bleeding, pelvic pain, bulk-related pressure symptoms, reproductive dysfunction, and infertility [5]. In contrast, FAs are the commonest breast neoplasms, occurring in 10% of young women and detected in 7~13% of screened populations, with peak incidence between 15 and 35 years [6,7]. They typically present as painless, mobile nodules and are often managed conservatively.

Both tumors expand during estrogen- and progesterone-rich states (reproductive years, pregnancy) and regress after menopause, indicating steroid–hormone dependence [8,9]. Despite this endocrine overlap, malignant progression is rare: uterine leiomyosarcomas arise de novo from myometrial cells rather than from pre-existing fibroids [10], and only a minority of FAs evolve into phyllode tumors or confer a modest increase in breast cancer risk [11]. In a previously published study, our research team reported that alterations affecting TP53, RB1, PTEN, MED12, YWHAE, and VIPR2 were present in most malignant smooth muscle tumors and uterine leiomyosarcoma [5]

At the genomic level, ULs and FAs converge on hotspot missense mutations in MED12 exon 2 (codon 44), found in ~70% of UL and ~60% of FA [12,13]. MED12 encodes a key subunit of the Mediator kinase module; its disruption is thought to perturb RNA-polymerase II transcriptional control, promoting mesenchymal proliferation. Beyond this shared driver, ULs are genomically heterogeneous: mutually exclusive subgroups exhibit HMGA2 or HMGA1 rearrangements, biallelic loss of FH, or recurrent deletions on chromosome 7q, each associated with distinct gene-expression profiles and chromosomal instability [14,15,16]. By contrast, multiple comparative-genomic-hybridization series have demonstrated that most FAs are karyotypically quiet apart from the initiating MED12 lesion [17].

Although these observations suggest that a common molecular spark ignites tissue-specific evolutionary programs, no study has yet mapped UL and FA genomes side-by-side within matched cohorts. We hypothesize that, after MED12 mutation, uterine smooth muscle and breast stromal cells encounter distinct selective pressures that yield divergent patterns of mutational processes, copy number change, and secondary drivers. By deploying whole-exome sequencing of paired UL and FA specimens, we provide new clarity on the molecular relationship between uterine fibroids and breast fibroadenomas, with improved depth and comparative insight beyond prior reports.

## 2. Materials and Methods

From March 2017 to December 2019, tissue was collected from 15 patients who were diagnosed with uterine fibroids and underwent surgery according to the SOP of the Human Derivatives Bank with the approval of the Institutional Review Committee of St. Mary’s Hospital in Uijeongbu.

Tissue genomic DNA obtained from patients with uterine fibroids was extracted, and whole-exome sequencing (WES) was performed. Further, blood obtained from each patient was compared with a reference genome.

For the generation of standard exome capture libraries, we used the Agilent SureSelect Target Enrichment protocol for the Illumina paired-end sequencing library (Version C2, December 2018) together with 1 ug input gDNA. In all cases, the SureSelect Human All Exon V6 probe set was used. Quantification of DNA and determination of DNA quality were measured by PicoGreen and agarose gel electrophoresis, respectively. We used 1 μg of each cell line’s genomic DNA diluted in EB buffer and sheared to a target peak size of 150–200 bp using the Covaris LE220 focused-ultrasonicator (Covaris, Woburn, MA, USA) according to the manufacturer’s recommendations. The 8 microTUBE Strip was loaded into the tube holder of the ultrasonicator and the DNA was sheared using the following settings: mode, frequency sweeping; duty cycle, 10%; intensity, 5; cycles per burst, 200; duration, 60 s × 6 cycles; and temperature, 4–7 °C. The fragmented DNA was repaired, an “A” ligated to the 3′ end, and Agilent adapters were ligated to the fragments. Once ligation had been assessed, the adapter-ligated product was PCR amplified. For exome capture, 250 ng of DNA library was mixed with hybridization buffers, blocking mixes, RNase block, and 5 µL of SureSelect all exon capture library, according to the standard Agilent SureSelect Target Enrichment protocol. Hybridization to the capture baits was conducted at 65 °C using a heated thermal cycler lid option at 105 °C for 24 h on a PCR machine. The captured DNA was washed and amplified. The final purified product was quantified using qPCR according to the qPCR Quantification Protocol Guide (KAPA Library Quantification kits for Illumina Sequencing platforms) and qualified using the TapeStation DNA screentape D1000 (Agilent, Santa Clara, CA, USA). Finally, we sequenced the data using the HiSeq™ 2500 platform (Illumina, San Diego, CA, USA).

The study protocol was approved by the Institutional Review Committee of St. Mary’s Hospital in Uijeongbu and was conducted in accordance with the standard operating procedures (SOP) of the Human Derivatives Bank. DNA was extracted from tissue samples collected from 15 patients diagnosed with uterine leiomyomas who underwent surgery between March 2017 and December 2019 at St. Mary’s Hospital in Uijeongbu, South Korea.

Seven fibroadenomas were composed of Chinese individuals from the European Nucleotide Archive (ENA) database (https://www.ebi.ac.uk/ena/browser/view/PRJEB5763 accessed on 7 June 2025).

Genomic DNA from 15 tumor samples and their matched blood samples from St. Mary’s Hospital were sent for WES. The sequencing reads were mapped to the hg38 reference genome using BWA-MEM. The sequencing data were processed using the GATK Best Practice workflow [18,19,20]. Conpair was used to confirm tumor-normal concordance for all samples, ensuring that the tumor and normal samples were correctly matched [21]. To determine the ancestry of individual samples within the cohort, Somalier ancestry analysis was used [22]. Somatic SNVs were called using GATK4 MuTect2 [23] and INDELs were called using the intersection of the two callers, MuTect2 and Strelka2 [24]. To remove common somatic variants, those with a minor allele frequency (MAF) greater than 0.1% in ExAC, gnomAD, and Bravo were excluded. Mutational matrices were constructed using SigProfilerMatrixGenerator (v1.2.31) [25], and mutational signatures were extracted using SigProfiler (v0.1.9) [26].

Somatic copy number variations (CNVs) were identified from 22 paired whole-exome sequencing (WES) samples, including 15 uterine leiomyomas and 7 fibroadenomas, using FACETS [27]. To calculate significantly amplified or deleted regions, enrichment analysis was performed using GISTIC2.0 [28]. The resulting chromosome-level segmentation data (genomic intervals), together with the location of the exome probes, were then analyzed in GISTIC to generate the copy number results. GISTIC was run with parameters of logR threshold for calling a segment amplified of ±0.25 and capping logR values at 1.5.

Microsatellite instability was analyzed using MSIsensor2 (https://github.com/niu-lab/msisensor2 accessed on 7 June 2025) in 15 uterine leiomyomas and 7 fibroadenomas.

## 3. Results

### 3.1. Sequencing Quality and Cohort Characteristics

We analyzed 15 uterine leiomyomas (ULs) and 8 publicly available breast fibroadenomas (FAs) with matched normal tissue (peripheral blood) from the same patients, enabling confident distinction between somatic and germline variants. Principal Component Analysis of tumor germline variants confirmed that all 15 UL patients and 7 of 8 FA patients clustered with the East Asian (EAS) population, while one FA sample (FA-008) clustered with the South Asian (SAS) population and was excluded to maintain genetic homogeneity (Figure S1). The final cohort comprised 15 UL and 7 FA samples, all of East Asian ancestry. High-quality whole-exome sequencing yielded mean target coverage at a 20× depth of 94.5% for ULs and 88.7% for FAs, both sufficient for reliable variant detection (Table 1). The slightly higher UL coverage indicates marginally greater statistical power for detecting low-frequency variants in these samples. This makes any findings of higher mutational burden in FAs more robust, as they would represent true biological differences rather than technical artifacts (Table 1). All tumors were histologically confirmed as benign, with no sarcomatous features in ULs and all breast lesions diagnosed as fibroadenomas rather than phyllodes tumors.

### 3.2. Somatic Mutation Burden and Tumor Purity

**Figure 1:**
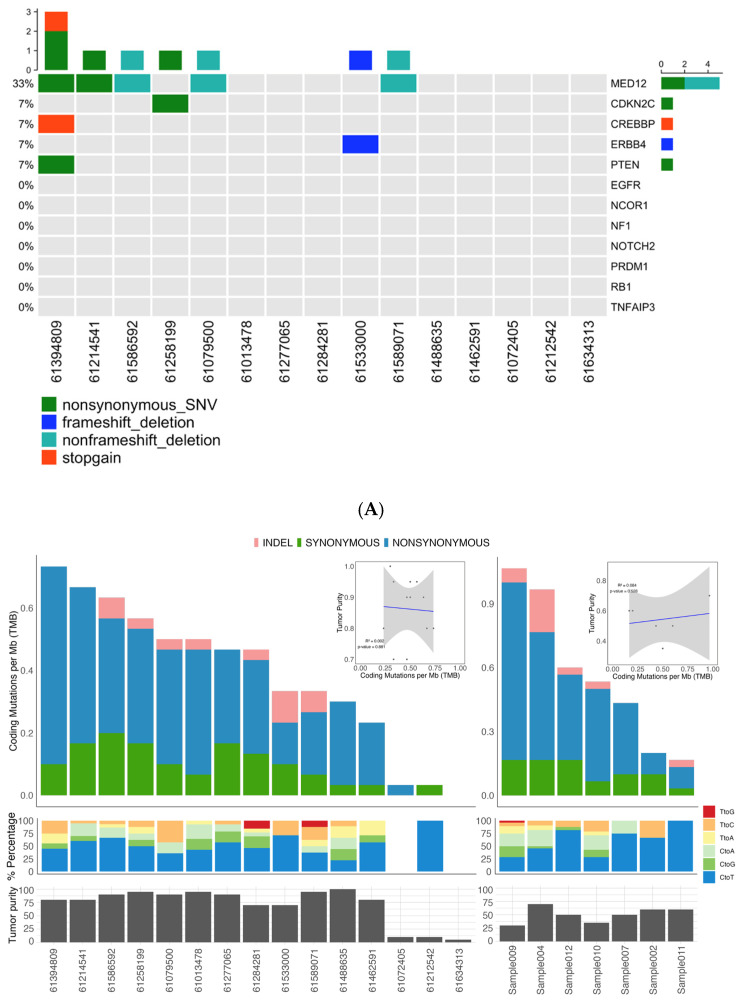

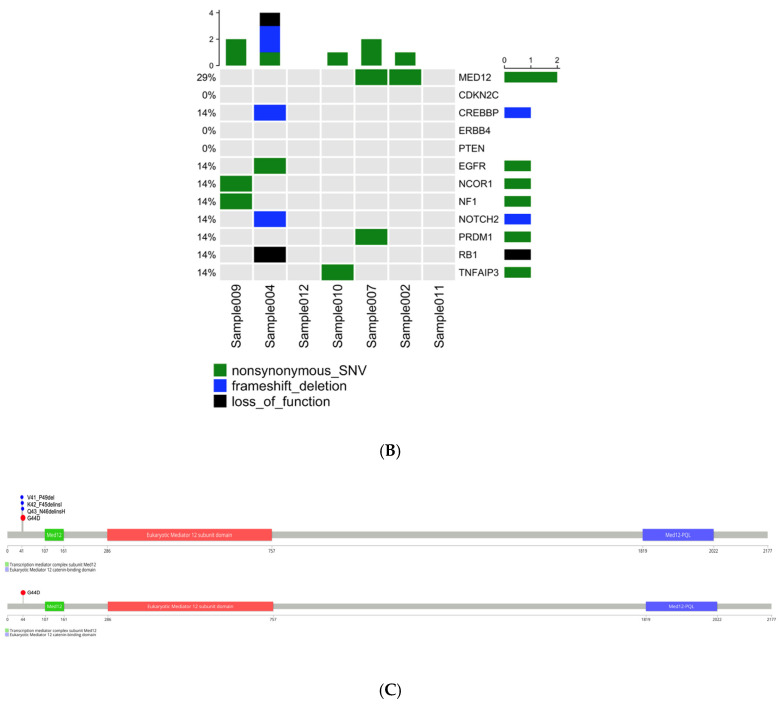
Somatic mutation landscape underlying uterine leiomyoma and fibroadenoma. (**A**) Distribution of somatic mutation in 15 uterine leiomyoma and 7 fibroadenoma samples. (**B**) Frequency and type of somatic mutations. Columns represent 15 uterine leiomyoma (**left**) and 7 fibroadenoma samples (**right**), and rows indicate cancer-related genes. (**C**) Schematic representation of SNVs and INDELs identified in the MED12 gene.

A total of 174 somatic mutations were called (mean = 11.60 per patient, median = 14.00 per patient) in ULs and 114 were called in FAs (mean = 16.86 per patient, median = 15.00 per patient) (Table 2). There was no correlation between tumor purity and somatic mutation burden in both tumors (R2 = 0.014, *p* = 0.67, Figure 1A). The median NS/S ratio was for ULs with a mean of 3.50, while the FAs had a median of 3.25 with a mean of 3.14 (Table 1). The median transition/transversion (Ti/Tv) ratio for ULs was 1.80 for ULs with a mean of 1.98, while the FAs had a median of 2.10 with a mean of 2.04 (Table 2). Analysis of tumor purity and mutational burden revealed an unexpected inverse relationship between these parameters (Figure 1A). ULs showed high mean tumor purity (0.86) but modest somatic variant counts (median of 14.00 per tumor), while FAs demonstrated significantly lower purity (0.51) yet harbored approximately 1.47-fold more somatic variants than ULs (median of 16.86 per tumor), though this difference did not reach statistical significance (Wilcoxon rank-sum test, *p*-value = 0.39). This paradoxical finding—higher mutations despite greater normal tissue dilution—cannot be explained by technical factors. The slightly lower exome coverage in FAs (88.7% vs. 94.5%) would bias toward variant under-detection rather than over-detection.

To ensure this high mutational load was not a consequence of widespread genomic instability caused by defective mismatch repair (MMR), we analyzed all samples for microsatellite instability (MSI) using MSIsensor2. All tumors in both cohorts were classified as microsatellite stable (MSS), ruling out MMR deficiency as the primary driver of this phenomenon (Figure S2).

This evidence strongly suggests that FAs are characterized by a “mutator phenotype,” a state in which the cellular machinery for maintaining genomic fidelity is compromised, leading to an accelerated accumulation of point mutations. This contrasts with the more modest mutational burden in ULs, suggesting their growth is likely driven by different mechanisms.

### 3.3. Shared MED12 Initiation with Divergent Secondary Driver Landscapes

Analysis confirmed MED12 mutations as a common initiating event in both tumor types, identified in 5/15 (33%) UL and 2/7 (29%) FA samples. Critically, both tumor types harbored the identical missense mutation c.131G > A (p.Gly44Asp) in exon 2, a known hotspot that disrupts MED12-Cyclin C binding and impairs CDK8/19 kinase activity within the Mediator transcriptional complex (Figure 1B). This shared mutation represents convergent molecular initiation between these distinct benign tumors.

Beyond MED12, the mutational landscapes diverged markedly. UL samples harbored secondary mutations in established tumor suppressors (CDKN2C: p.L21H, PTEN: p.S229L) and the transcriptional coactivator CREBBP (p.C2725T), none of which appeared in FA samples (Figure 1B). Conversely, one FA sample (FA-004) exhibited an exceptional molecular profile: four loss-of-function mutations (p.G1469Afs*9, pK849Rfs*6, pK63X) coupled with a gain-of-function mutation (p.T218P) in EGFR (Figure 1B). This constellation of mutations, particularly the oncogenic EGFR alteration typically associated with aggressive malignant breast cancers, contrasts starkly with the sample’s benign histological classification.

### 3.4. Dichotomous Genomic Stability: CNA-Driven ULs vs. CNA-Quiet FAs

**Figure 2:**
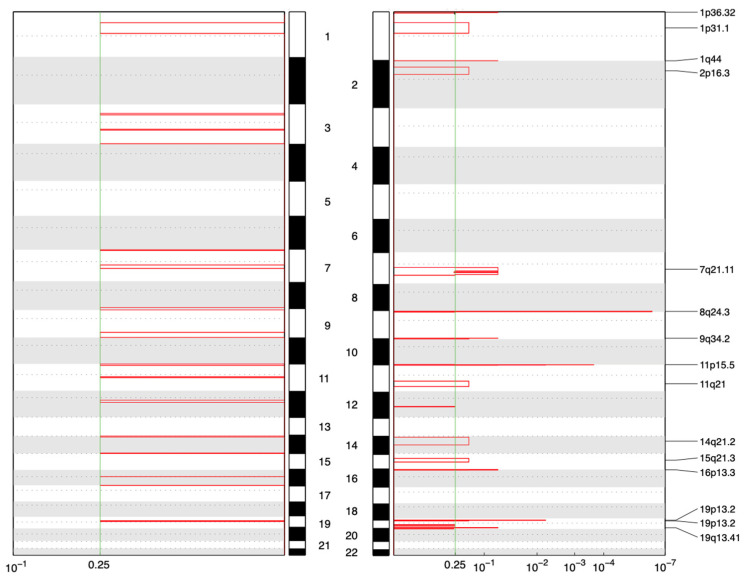
GISTIC-based analysis of recurrent CNV alterations (q-value threshold: 0.25), showing significant copy number amplifications in 7 samples (**left**) and 15 UL samples (**right**).

Copy number analysis revealed fundamentally divergent evolutionary strategies between the two tumor types. All 15 uterine leiomyomas (ULs) exhibited somatic copy number amplifications (total of 15 distinct events), with 6/15 (40%) encompassing known oncogenes. Notable examples included recurrent 12q15 amplifications encompassing HMGA2, consistent with the established HMGA2-overexpression pathway in non-MED12-mutant fibroids. Additional amplifications involving HMGA1 (6p21), EGFR (7p), and regions harboring other growth-promoting genes (Figure 2). These findings align with the recognized molecular subclasses of UL (MED12-mutant, HMGA2/HMGA1-upregulated, FH-inactivated, and COL4A5/6-deleted) and demonstrate chromosomal instability as a key driver mechanism. 

In complete contrast, none of the seven fibroadenomas (FA) demonstrated any detectable copy number alterations. Even the hypermutated FA-004 sample, despite harboring multiple driver mutations including EGFR gain of function, maintained a flat copy number profile. This genomic quiescence confirms that FAs do not require large-scale structural alterations for tumorigenesis and distinguishes them from phyllodes tumors, which typically acquire CNAs involving oncogenes (EGFR, PDGFB) or tumor suppressors (TP53, RB1).

This dichotomy defines two distinct evolutionary strategies: ULs employ “macro-evolution” through chromosomal instability, efficiently altering gene dosage of multiple loci simultaneously. In contrast, FAs utilize “micro-evolution,” maintaining chromosomal integrity and acquiring drivers through sequential point mutations.

### 3.5. Mutational Signatures Reveal Distinct Endogenous Mutagenic Processes

**Figure 3:**
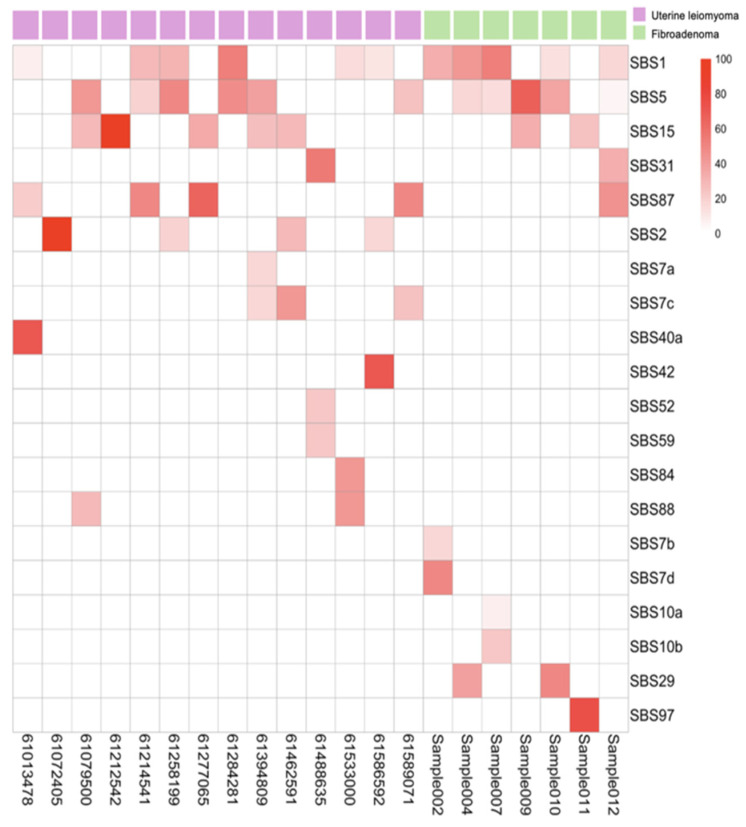
Heatmap of mutation signatures identified using SigProfilerAssignment. Columns represent 15 uterine leiomyoma (pink) and 7 fibroadenoma (green) samples, and rows correspond to individual mutation signatures.

Mutational signature analysis using COSMIC v3 revealed shared baseline processes and distinct tumor-specific mutagenic mechanisms. Both tumor types exhibited ubiquitous clock-like signatures SBS1 (5-methylcytosine deamination) and SBS5 (age-related), along with low-level SBS15 (MMR-associated) despite microsatellite stability, indicating background aging processes with possible minor MMR impairment (Figure 3).

The tumors diverged markedly in their specific mutational processes. ULs displayed SBS2 (APOBEC activity), SBS42 (haloalkane exposure), SBS84 (AID activity), and SBS88 (colibactin exposure), suggesting episodic enzymatic DNA damage while maintaining replication fidelity (Figure 3). In contrast, FAs exhibited a distinct mutator phenotype characterized by defective DNA polymerase epsilon proofreading (SBS10a, SBS10b) and associated with environmental mutagenic exposures such as tobacco chewing (SBS29) and ultraviolet light (SBS7b, SBS7d) (Figure 3). This fundamental difference—intact replication machinery in ULs versus error-prone replication with multiple mutagenic sources in FAs—mechanistically explains why FAs accumulate more mutations despite lower tumor purity. It establishes their divergent evolutionary pathways of chromosomal instability versus point mutation accumulation.

## 4. Discussion

This study provides the first direct genomic comparison of ULs and FAs, revealing convergent initiation through MED12 mutations but fundamentally divergent evolutionary trajectories. The identification of identical MED12 p.G44D mutations in both tumor types confirms a shared molecular origin, establishing disruption of the Mediator kinase module as a common vulnerability in hormone-responsive mesenchymal tissues. It is well established that MED12 is involved in Wnt signaling and the function of the Mediator complex [29,30]. However, post-initiation evolution follows distinct pathways: UL evolves through chromosomal instability with frequent copy number amplifications affecting oncogenes. In contrast, FA follows a mutator phenotype characterized by defective DNA polymerase proofreading while maintaining chromosomal stability.

The dichotomous evolution likely reflects intrinsic differences in cellular tolerance for genomic alterations. Uterine smooth muscle cells appear to tolerate aneuploidy and large-scale structural changes, evidenced by recurrent amplifications involving HMGA2, HMGA1, and other growth regulators, yet remain histologically benign. This suggests robust tissue-specific constraints preventing malignant transformation despite chromosomal chaos. Conversely, breast stromal cells maintain chromosomal integrity but accumulate point mutations through compromised replication fidelity (POLE defects), indicating selection against structural alterations while permitting hypermutation.

The discovery of an EGFR gain-of-function mutation alongside multiple loss-of-function mutations in a histologically benign FA challenges traditional benign–malignant classifications. This molecular profile, typically associated with aggressive malignancies, suggests that histology alone may be insufficient for risk assessment. While ULs rarely progress to leiomyosarcomas regardless of accumulated alterations, FAs may represent a continuum toward phyllodes tumors when acquiring specific driver combinations. This fundamental difference has important implications for clinical management.

Our findings support a model where benign tumors exist on a genomic spectrum rather than as categorically distinct entities. The shared MED12 driver demonstrates convergent selection in hormone-responsive tissues, while divergent secondary events reflect tissue-specific evolutionary constraints and mutational processes. The FA-specific polymerase proofreading defects indicate additional layers of genomic instability absent in ULs.

These insights argue for incorporating molecular profiling into clinical decision making for a subset of benign lesions. While most FAs and ULs can be managed conservatively, those harboring high-risk molecular features may warrant enhanced surveillance or intervention. Future studies should validate these findings in larger cohorts and functionally characterize the identified mutations. They should also prospectively follow patients with molecularly high-risk benign tumors to quantify progression risk and develop evidence-based management guidelines. Finally, it will be important to experimentally validate that the two benign tumors follow distinct evolutionary paths.

## 5. Conclusions

Whole-exome sequencing of matched ULs and FAs revealed shared MED12 mutations as a common initiating event, yet fundamentally different evolutionary trajectories. Both tumor types harbored identical MED12 p.G44D mutations, confirming mediator complex dysfunction as a unifying driver in hormone-responsive tissues. However, their downstream genomic architecture diverged markedly: UL exhibited chromosomal instability with frequent copy number amplifications affecting oncogenes, while FA maintained chromosomal stability but accumulated excess point mutations through defective DNA polymerase proofreading and enzymatic mutagenesis.

Despite these differences, both tumor types remained microsatellite stable with relatively low overall mutation burdens, consistent with benign behavior. The notable exception was one FA harboring an EGFR gain-of-function mutation alongside multiple loss-of-function mutations—a molecular profile typically associated with malignancy despite benign histology. This finding challenges traditional classification systems and suggests that genomic profiling may identify high-risk lesions requiring enhanced surveillance. In contrast, UL tolerated multiple chromosomal alterations without malignant progression, indicating tissue-specific constraints on transformation.

These results demonstrate that identical driver mutations yield distinct genomic outcomes depending on cellular context. UL evolution proceeds through chromosomal instability, potentially involving HMGA2 upregulation and other large-scale events, while FA evolution relies on point mutation accumulation with selection against structural alterations except during phyllodes transition. This comparative analysis establishes that while these common benign tumors share a genetic foundation, they navigate tumorigenesis through divergent genomic routes. These insights may inform risk stratification and targeted therapeutic approaches for hormone-responsive benign neoplasms.

## Figures and Tables

**Table 1:** Sequencing statistics.

Characteristics	Uterine Leiomyoma				Fibroadenoma			
	15 Tumor Samples		15 Normal Samples		8 Tumor Samples		8 Normal Samples	
	Mean	Median	Mean	Median	Mean	Median	Mean	Median
Number of reads (M)	145.6	149.8	79.5	68.2	186.63	173.55	161.98	162.7
Mean coverage (X)	139.4	142.9	76.3	66	148.18	140.5	132.21	139.1
Median coverage (X)	121.1	122	65.8	57	123.63	117	108.5	116
Percent of bases covered at least 8X	96.00%	96.00%	95.00%	94.70%	92.73%	92.70%	92.28%	92.30%
Percent of bases covered at least 20X	94.50%	94.50%	90.00%	88.90%	88.77%	88.75%	87.96%	88.30%

**Table 2:** Somatic mutation QC.

		UL	FA
Total somatic mutation	mean	11.60	16.86
	median	14.00	15.00
NS/S ratio	mean	3.22	3.14
	median	3.50	3.25
Ti/Tv ratio	mean	1.98	2.04
	median	1.80	2.10
Tumor purity	mean	0.86	0.51
	median	0.90	0.50

## Data Availability

The data presented in this study are available in in K-BDS at https://www.kbds.re.kr reference number KAP241746.

## Supplementary Materials

The following supporting information can be downloaded at https://www.mdpi.com/article/10.3390/jcm14207325/s1.
