# Supplementary material for: Tissue-Specific Genomic Evolution Despite Shared MED12 Mutations in Benign Tumors
Source: J Clin Med. 2025 Oct 16;14(20):7325. doi: 10.3390/jcm14207325 (PMC12565393; doi:10.3390/jcm14207325)
Supplement: Supplementary file 1 [file jcm-14-07325-s001.zip › jcm-3750286-supplementary.pdf]

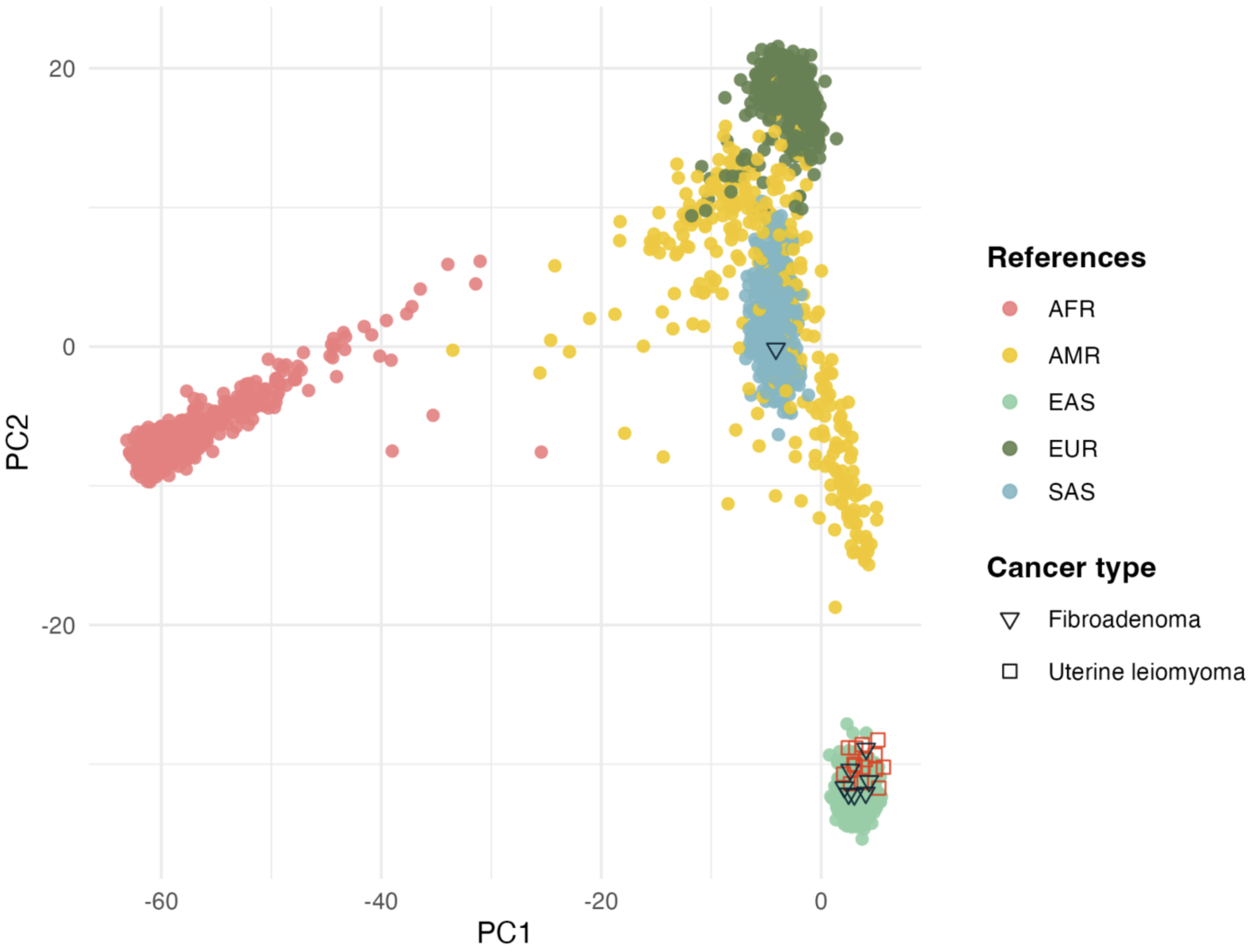

Figure S1. PCA plot of population genetic structure based on Somalier ancestry analysis, including 15 uterine leiomyoma and 7 fibroadenoma samples

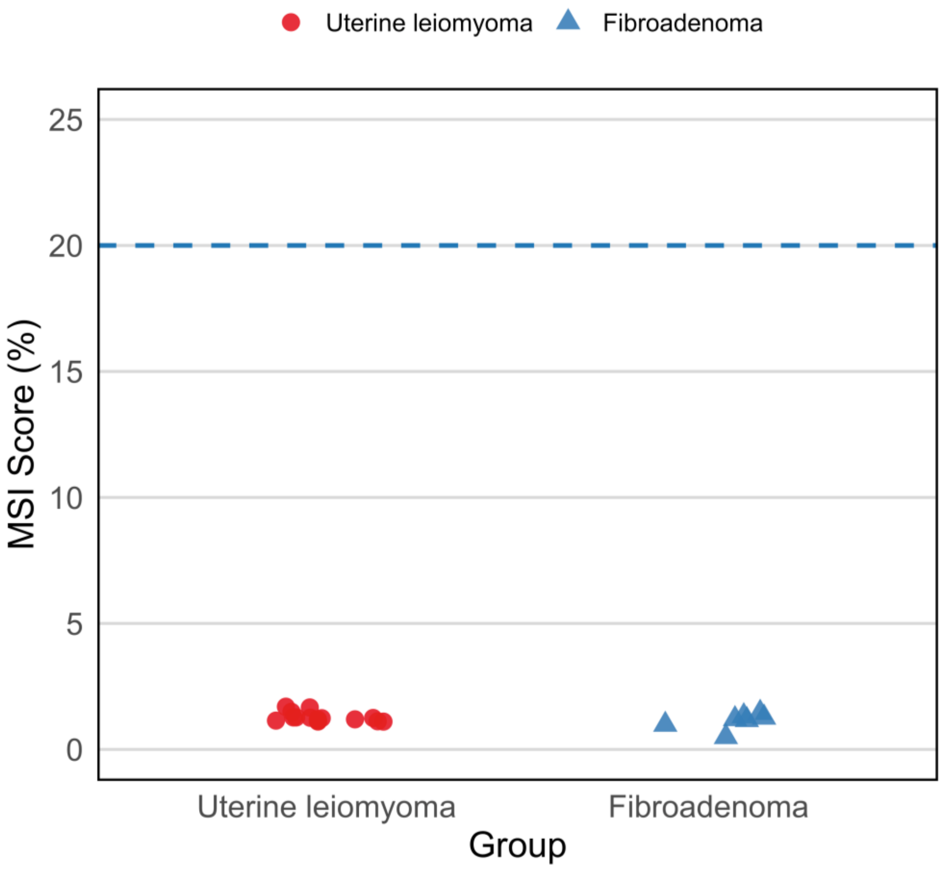

Figure S2. Dot plot showing MSI scores calculated using MSIsensor2. The blue line indicates the threshold for microsatellite instability (MSI score ≥ 20%).

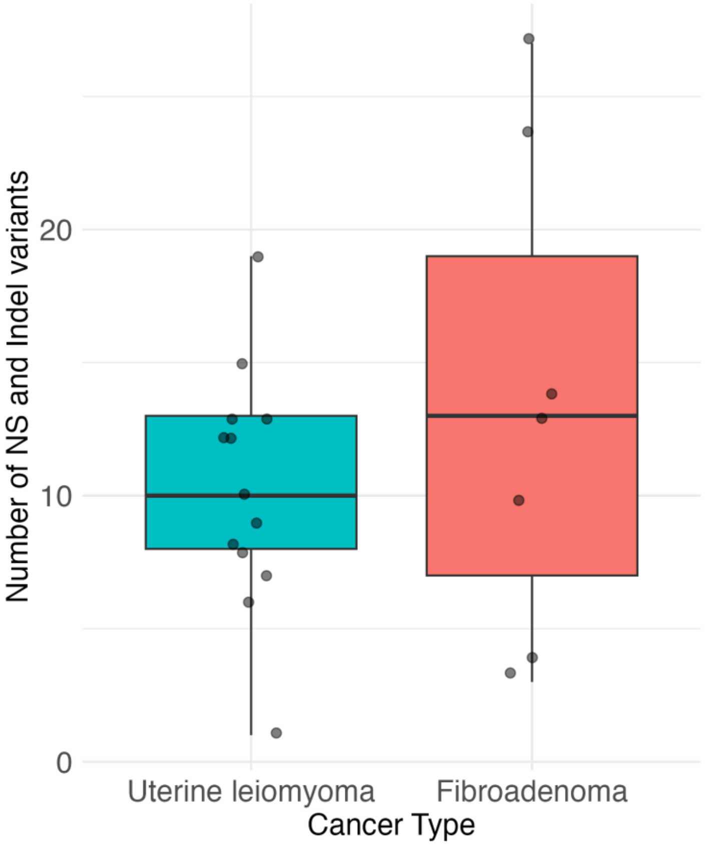

Figure S3. Boxplot showing indel and nonsynonymous variant counts in fa and ul samples

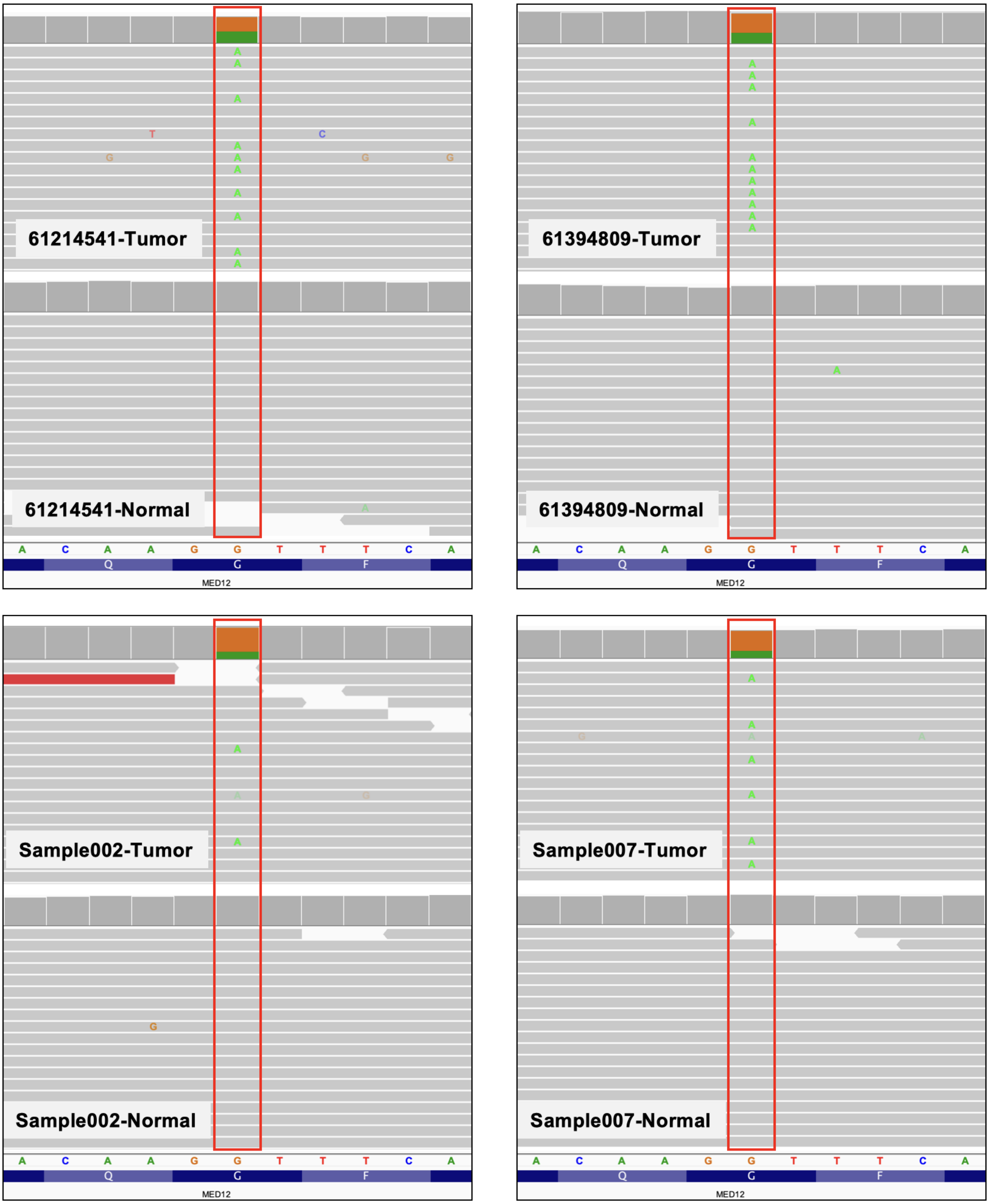

Figure S4. IGV tracks showing the MED12 p.G44D mutation site (c.131G>A) in representative samples from both tumor types.
